# Simple and Divided Leaves in Ferns: Exploring the Genetic Basis for Leaf Morphology Differences in the Genus *Elaphoglossum* (Dryopteridaceae)

**DOI:** 10.3390/ijms21155180

**Published:** 2020-07-22

**Authors:** Alejandra Vasco, Barbara A. Ambrose

**Affiliations:** 1 Botanical Research Institute of Texas, 1700 University Drive, Fort Worth, TX 76107-3400, USA; 2 The New York Botanical Garden, 2900 Southern Blvd, Bronx, NY 10458-5126, USA

**Keywords:** *Class I KNOX*, Dryopteridaceae, *Elaphoglossum*, ferns, fronds, leaf diversity, leaf evolution and development, megaphyll

## Abstract

Despite the implications leaves have for life, their origin and development remain debated. Analyses across ferns and seed plants are fundamental to address the conservation or independent origins of megaphyllous leaf developmental mechanisms. *Class I KNOX* expression studies have been used to understand leaf development and, in ferns, have only been conducted in species with divided leaves. We performed expression analyses of the *Class I KNOX* and *Histone H4* genes throughout the development of leaf primordia in two simple-leaved and one divided-leaved fern taxa. We found *Class I KNOX* are expressed (1) throughout young and early developing leaves of simple and divided-leaved ferns, (2) later into leaf development of divided-leaved species compared to simple-leaved species, and (3) at the leaf primordium apex and margins. *H4* expression is similar in young leaf primordia of simple and divided leaves. Persistent *Class I KNOX* expression at the margins of divided leaf primordia compared with simple leaf primordia indicates that temporal and spatial patterns of *Class I KNOX* expression correlate with different fern leaf morphologies. However, our results also indicate that *Class I KNOX* expression alone is not sufficient to promote divided leaf development in ferns. *Class I KNOX* patterns of expression in fern leaves support the conservation of an independently recruited developmental mechanism for leaf dissection in megaphylls, the shoot-like nature of fern leaves compared with seed plant leaves, and the critical role marginal meristems play in fern leaf development.

## 1. Introduction

Leaves are the dominant organ in most extant vascular plants and their evolutionary origin, likely in the early Devonian, fundamentally changed not only life on earth, but also the basic Bauplan of vascular plants [1,2]. Despite this profound importance, the number of times leaves have evolved in vascular plants is still debated, and it is mainly within Euphyllophytes (ferns and seed plants) that the number of times leaves have evolved is still not settled. In Euphyllophytes, leaves have been hypothesized to have evolved from one up to nine times [3,4,5,6]. Particularly in the ferns, there is currently no consensus on whether the leaves of major lineages such as the Equisetaceae (horsetails), Psilotaceae (whisk ferns), Ophioglossaceae, Marattiaceae, and the leptosporangiate ferns are homologous [7,8]. 

The leaves of the Euphyllophytes are also called megaphylls and are characterized by an enormous morphological diversity; they can be simple, lobed, pedate, digitate, or divided (also termed compound or dissected). The genetic developmental basis of this enormous diversity has been mainly studied in model angiosperms, but it is largely unknown in ferns. Comparative analyses across Euphyllophyte lineages are essential to gain the comparative data needed to resolve the long-standing questions of leaf evolution and development. Comparative approaches to understand the genetic pathway affecting megaphyll shape outside of angiosperms have mainly focused on the *Class I KNOTTED1-like HOMEOBOX* (*Class I KNOX*) genes [9,10,11].

In angiosperms, genetic studies have explored the basis for differences in leaf division in the species with simple leaves: *Arabidopsis thaliana*, *Zea mays*, and *Antirrhinum majus,* compared with species with divided leaves: *Cardamine hirsuta*, *Lycopersicon esculentum*, *Pisum sativum*, and *Medicago truncatula* [12,13,14]. In angiosperms, Class I KNOX proteins are generally necessary for meristem maintenance and are expressed throughout the vegetative and floral shoot apical meristems (SAMs) and down-regulated in leaf primordia and floral organs [14,15,16,17,18,19,20,21]. In angiosperms with simple leaves, Class I KNOX proteins are expressed in the SAM, and down-regulated in incipient leaf primordia and throughout primordium development [14,17,20]. In many angiosperms with divided leaves, Class I KNOX proteins are also expressed in the SAM and down-regulated in incipient leaf primordia; however, they are expressed later in young leaf primordia and in sites of leaflet initiation [9,22]. Class I KNOX expression and function in leaves have been shown to underlie divided leaf morphology in angiosperms [9,22,23]. In angiosperms with divided leaves, over-expression of Class I KNOX produces mature leaves that are highly divided [24,25,26]. Meanwhile, in plant species with simple leaves, an overexpression of *Class I KNOX* genes does not result in divided leaves, but leaves with lobes or crenulated margins [17,24,27,28]. Therefore, *Class I KNOX* genes are required, but not sufficient, to produce divided leaves in angiosperms [9,29].

In gymnosperms, *Class I KNOX* expression has been reported for *Picea abies* (simple leaves) [30], *Zamia floridans* (divided leaves) [9], and *Welwitschia mirabilis* (simple leaves) [31]. These studies have shown that *Class I KNOX* are expressed in the SAM and down-regulated in incipient leaf primordia of simple and divided gymnosperms leaves, and up-regulated in divided leaved species. These patterns of expression are similar to those found in angiosperms, providing support for the homology of seed plant leaves.

For ferns, the expression profiles of *Class I KNOX* genes have been studied only in the species with divided leaves: *Osmunda regalis* [10], *Anogramma chaerophylla* [9], *Ceratopteris richardii* [32], and *Elaphoglossum peltatum* f. *peltatum* [33]. These studies showed that *Class I KNOX* genes are expressed in the fern’s SAM, in young leaf primordia, and in the margins of old leaf primordia, similar to seed plants; but unlike in most seed plants, *Class I KNOX* genes were found not to be down-regulated in incipient leaf primordia of ferns with divided leaves [9,32]. This lack of down-regulation has been interpreted either as leaves of ferns and seed plants having evolved independently [9], or as a reflection of the delayed determinacy (i.e., persistent meristematic activity) exhibited by fern leaves [10]. However, *Class I KNOX* expression in the margins of old leaf primordia in fern species with divided leaves suggests that the same network for divided leaf development might be conserved in ferns and seed plants [9,10,32]. Central to resolving this debate are ferns with simple leaves, whose *Class I KNOX* expression patterns have not been studied before. The expression of *Class I KNOX* in simple leaves in ferns will help to better understand if the differences in expression found between ferns and angiosperms are linked to leaf morphology or if they can explain the different evolutionary origins of fern and seed plants leaves. Such a comparative approach is fundamental to address questions about the conservation or independent origins of megaphyllous leaf developmental mechanisms in plants.

**Figure 1:**
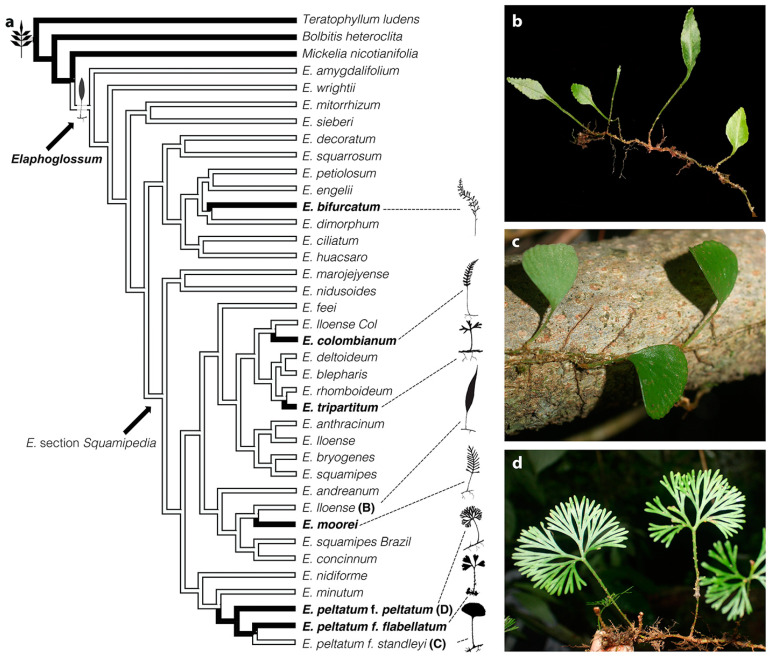
Leaf morphological variation in the genus *Elaphoglossum.* (**a**) Phylogeny of *Elaphoglossum* with leaf dissection optimized onto the tree (characters were optimized under a parsimony criterium with Mesquite V. 3.5). Black branches = divided leaves, white branches = simple leaves (modified from [36]). Divided-leaf taxa are in bold and displayed as shadow diagrams (not to scale), and the letter after species indicates species included in the expression studies. (b–d) The three closely related taxa studied of *Elaphoglossum* section *Squamipedia*. (**b**) *Elaphoglossum lloense* (simple leaves). (**c**) *Elaphoglossum peltatum* f. *standleyi* (simple leaves). (**d**) *Elaphoglossum peltatum* f. *peltatum* (divided leaves).

Among leptosporangiate ferns, *Elaphoglossum* is one of the most diverse genera of ferns and its nearly 600 species are characterized by simple entire leaves [34]. There are only six species of *Elaphoglossum* that have divided leaves and four of them belong to a monophyletic group of 20 species, *Elaphoglossum* section *Squamipedia* [35,36,37]. The species with divided leaves belonging to section *Squamipedia* are *E. colombianum* (Maxon) Mickel, *E. moorei* (E. Britton) Christ, *E. peltatum* (Sw.) Urban, and *E. tripartitum* (Hook. & Grev.) Mickel (Figure 1a). Phylogenetic molecular studies have shown that, within section *Squamipedia*, the four species with divided leaves are not monophyletic and instead have had independent evolutionary origins from ancestors with simple, entire leaves [36] (Figure 1a). This suggests that the four species with divided leaves in section *Squamipedia* represent four independent reversions or new acquisitions of the divided condition [36,38], providing a fascinating system to study the evolution and development of leaf division in ferns within a robust phylogenetic framework.

To better understand the genetic and developmental basis underlying fern leaf morphological diversity and to compare this with what is known for ferns and seed plants, we isolated *Class I KNOX* orthologs from ferns, investigated their evolution, and studied their expression in three taxa with different leaf morphologies belonging to *Elaphoglossum* section *Squamipedia* (Figure 1). We also used the expression of *Histone H4* to better understand the leaf development of fern species with simple and divided leaves. *H4* genes have been previously used to assay cell-cycle activity in lateral organs and, as such, they can be used as a cellular division marker [22,39].

The selected three taxa were as follows: *Elaphoglossum lloense* (Hook.) T. Moore with simple, entire leaves, typical of most species within the genus (Figure 1b); *Elaphoglossum peltatum* f. *standleyi* (Maxon) Mickel with simple leaves that are circular to lunate (Figure 1c); and *E. peltatum* f. *peltatum*, with divided leaves cleaved medially into two halves, where the two halves are divided subdichotomously up to seven times (Figure 1d). *Elaphoglossum peltatum* and its forms (two of them included in this study, forma *standleyi* and forma *peltatum*) have perplexed taxonomists for years [36,40,41,42,43,44]. Several authors have considered these forms as different species and not as merely phenotypic variants, because they appear quite distinct from each other and may even grow intermixed and maintain their distinctness. However, recent studies considered them as different forms of the same species, because examination of herbarium specimens reveals many intermediates among all the forms and because phylogenetic studies recover all three forms as part of the same clade, but not reciprocally monophyletic [35,36]. 

By studying these three closely related fern taxa with different leaf morphologies, two with simple leaves, we wanted to better understand if the leaf developmental genes and their expression are conserved among ferns and angiosperms with different leaf morphologies, and if differences in the patterns of expression of *Class I KNOX* genes correlate with different fern leaf morphologies.

## 2. Results

### 2.1. Evolutionary History of Class I KNOX Transcription Factors in Ferns

**Figure 2:**
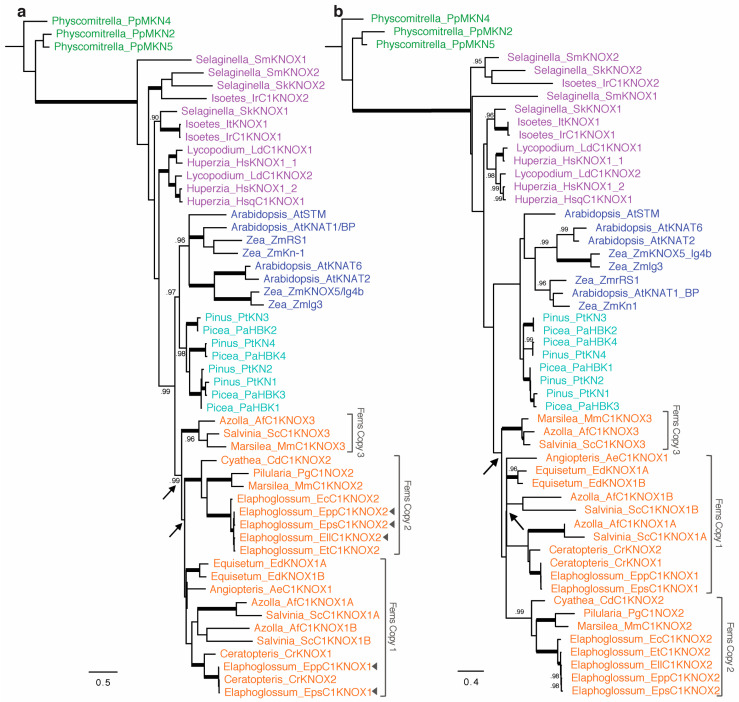
Phylogeny and evolution of *Class I KNOX* genes in ferns. (**a**,**b**) Phylograms inferred with (**a**) nucleotides and (**b**) amino acids presented as majority-rule consensus trees recovered in Bayesian inference (BI) analysis, including branch lengths and posterior probability (PP) values for nodes. Thick branches indicate PP = 1. PP values below 0.90 are not displayed. Colors of clade names correspond to the sources of the genes: green, bryophytes; purple, lycophytes; orange, ferns; light blue, gymnosperms; dark blue, angiosperms. Species abbreviations are listed in Appendix A. Arrowheads in (**a**), genes used for in situ hybridization analyses; arrows, inferred duplications within ferns.

To gain a more detailed evolutionary history of *Class I KNOX* in ferns and to discover the putative *Elaphoglossum Class I KNOX* gene copies for our expression studies, we isolated putative homologs from selected species spanning the phylogeny of ferns, and all three orders of lycophytes by PCR and database mining (Appendix A). We identified 13 new sequences by PCR. The final analyzed matrix included 53 sequences, of which 22 belonged to ferns. The aligned matrix had 732 nucleotide and 244 amino acid characters and included the four domains encoded by *KNOX* genes (KNOX1, KNOX2, ELK, and TALE-HD). The final data set is deposited in figshare (10.6084/m9.figshare.12576581). Analyses of the nucleotide and amino acid sequences yielded congruent tree topologies. The phylogenetic relationships found are presented as majority-rule consensus trees, including branch lengths and posterior probability values for nodes (Figure 2).

Lycophyte sequences are not recovered as monophyletic, but in four different clades successively sister to euphyllophytes. Each lineage of Lycophytes, Selaginellales, Lycopodiales, and Isoetales, has at least two copies of *Class I KNOX*. Fern sequences form a monophyletic group sister to all seed plant *Class I KNOX* genes. During the evolution of ferns, at least two major duplication events are inferred (Figure 2, arrows), thus ferns have at least three copies of *Class I KNOX* genes. One copy (Copy 3 in Figure 2), which is sister to the other two, consists exclusively of sequences from the heterosporous ferns (order Salviniales). The other two copies of ferns *Class I KNOX* are sister to each other and include all the major lineages of ferns. The two *Class I KNOX* copies previously reported for the fern *Ceratopteris richardii* (*CrKNOX1* and *CrKNOX2*; Sano et al., 2005) belong to Fern *Class I KNOX* Copy 1 (Figure 2).

For two of our study species, *Elaphoglossum peltatum* f. *peltatum* (divided leaves) and *E. peltatum* f. *standleyi* (simple leaves), our mining for *KNOX* genes using degenerate primers recovered Copy1 and Copy 2 of ferns *Class I KNOX* (*EppC1KNOX1*, *EppC1KNOX2, EpsC1KNOX1*, and *EpsC1KNOX2*, Figure 2); for *E. lloense* (simple leaves), we only recovered Copy 2 (*EllC1KNOX2*, Figure 2). We found that all these *Elaphoglossum Class I KNOX* genes are recovered in a clade sister to all the well-known *Class I KNOX* angiosperm genes (Figure 2). Comparison of both *Class I KNOX* copies does not indicate that they are differentially spliced (Figure S1).

### 2.2. Development of Simple and Divided Leaves of Elaphoglossum

One characteristic typical of most fern leaves is their coiled young emerging leaves. Those have been referred to as crosiers or fiddleheads. Just as the whole leaf is coiled in bud, so too are its subdivisions, the pinnae and/or pinnules. Presumably, the function of coiling is to protect the soft meristematic parts concealed within the fiddlehead. Fiddleheads are highly distinctive of ferns because they are absent from lycophytes and nearly all seed plants [7].

**Figure 3:**
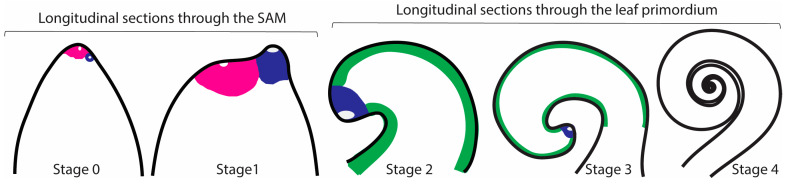
Stages of leaf development in the studied ferns and the meristems involved (pink = SAM, blue = LAM, green = MM). **Stage 0** (leaf initiation) Leaf initiation begins with the enlargement of an epidermal cell close to the shoot apical meristem. **Stage 1** (early leaf development): the leaf primordium is a protrusion that is more or less circular in outline; it has a prominent leaf apical initial (LAI). The LAI cuts off two files of cells that will become the marginal meristem (MM). Anatomically, the leaf apical meristem (LAM) resembles the shoot apical meristem (SAM). **Stage 2** (middle leaf development): the apex of the leaf primordium is clearly curved with an apparent LAI and MM. Basipetal procambium development is apparent. **Stage 3** (late leaf development): the apex of the leaf primordium apex is extremely curved towards the shoot apex owing to more cell divisions on the abaxial side. **Stage 4** (late crosier formation; pinna emergence): the crosier is apparent and the LAI is the same size as the rest of the cells and no longer dominant; in divided leaves, acropetal development of pinnae is apparent (not shown). Different stages not to scale. White regions in the SAM and LAM indicate leaf apical initial/s.

The three *Elaphoglossum* species studied here have long creeping stems (Figure 1b–d). Leaves are distichous (two vertical columns on opposite sides of the stem), alternate, and distant. Leaf primordia develop and grow slowly compared with the stem elongation rate, which is why in our studied species there is a relatively long distance among the visible developing leaves. In general, when plants are in the field growing with sufficient space and humidity, leaves only start to uncoil after the sixth visible leaf (Vasco, pers. Obs.). Delayed leaf expansion seems to be a characteristic of ferns with long creeping stems (Vasco, pers. Obs). Many studies of leaf development label leaves using plastochron numbers. Using a similar terminology for this study was not possible, mainly because of the delayed leaf expansion as described. Here, we defined five developmental stages based on leaf primordium morphology following our observations of histological sections and previously published fern leaf morphological and anatomical analyses [45,46,47,48,49] (Figure 3).

**Figure 4:**
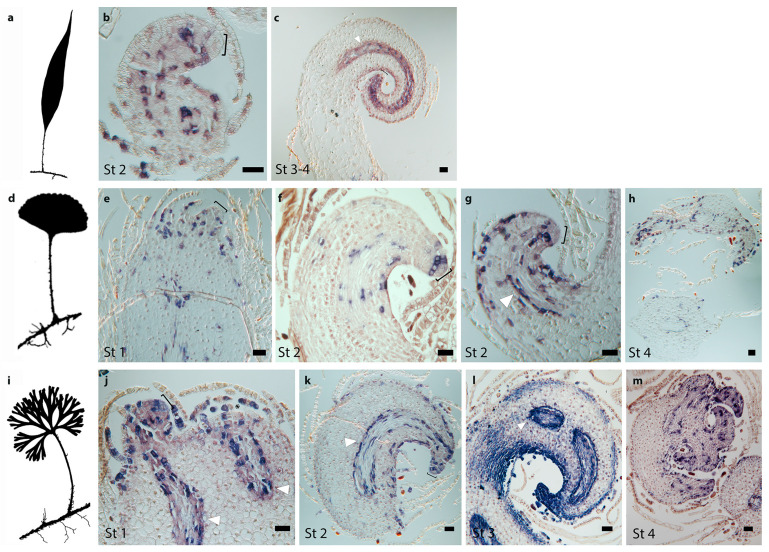
Cell division patterns as indicated by expression of *Histone H4* genes during leaf development in species of the fern genus *Elaphoglossum* with simple and divided leaves. (**a–c**) *Elaphoglossum lloense* (simple leaves). (**b,c**) Expression patterns of *ElH4*, longitudinal sections through the leaf primordium. (**b**) Expression in cells surrounding the leaf apical initial (LAI) and random cells throughout the primordium. (**c**) Expression in the apical region behind the LAI, in the procambium, and in the apical region of the margins. (**d–h**) *Elaphoglossum peltatum* f. *standleyi* (simple leaves). (**e–h**) Expression patterns of *EpsH4*. (**e–g**) Longitudinal sections through the shoot apical meristem (SAM) and/or leaf primordium. (**e**) Expression in random cells throughout the primordium that do not include the LAI. (**f**) Expression in the apical region right behind the LAI and in random cells throughout the primordium. (**g**) Expression in the apical region behind the LAI, in the procambium, and in cells of the margins. (**h**) Transverse section of old developing leaf, little expression in petiole and random expression throughout the lamina. (**i–m**) *Elaphoglossum peltatum* f. *peltatum* (divided leaves). (**j–m**) Expression patterns of *EppH4*. (**j–l**) Longitudinal sections through the SAM and/or leaf primordium. (**j**) Expression in the apical region including the LAI and in random cells throughout the primordium. (**k**) Expression in the apical region behind the LAI, in the procambium, and in cells of the margins. (**l**) Expression in the apical region, the procambium, and the margins; expression is discrete on the adaxial margin. (**m**) Transverse section of old developing leaf, little expression in petiole and random expression throughout the lamina. St, leaf developmental Stages following Figure 3; asterisk, SAM; brackets, LAI; white arrowhead, procambium. Bars = 40 um.

**Figure 5:**
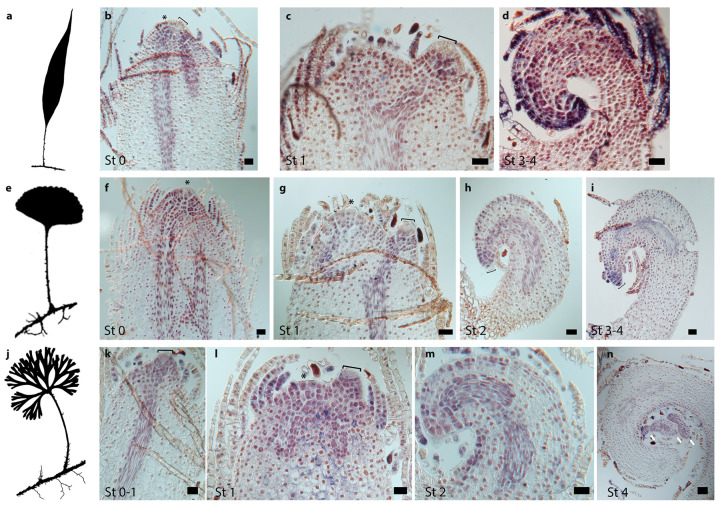
Expression patterns of *Class I KNOX* genes during leaf development in species of the fern genus *Elaphoglossum* with simple and divided leaves, longitudinal sections through the shoot apical meristem (SAM) and/or leaf primordia. (**a–d**) *Elaphoglossum lloense* (simple leaves). (**b–d**) Expression patterns of *EllC1KNOX2*. (**b**) Expression throughout the entire apical dome of the SAM and procambium, expression lacking from the incipient leaf primordium. (**c**) Expression throughout the entire young leaf primordium, lacking from the LAI. (**d**) Expression in the apical region, procambium, and the margins distally particularly abaxially. (**e–i**) *Elaphoglossum peltatum* f. *standleyi* (simple leaves). (**f–i**). Expression patterns in *E. peltatum* f. *standleyi* of (**f–h**) *EpsC1KNOX2* and (**i**) *EpsC1KNOX1*. (**f**) Expression throughout the entire apical dome of the SAM and procambium. (**g**) Expression in the SAM, procambium, and throughout the entire young leaf primordium, lacking from the LAI. (**h**) Expression in the apical region, procambium, and the margins, lacking from the LAI. (**i**) Expression in the apical region including the LAI and in the procambium; expression is absent from the margins. (**j–n**) *Elaphoglossum peltatum* f. *peltatum* (divided leaves). (**k–n**) Expression patterns of *EppC1KNOX2*. (**k**) Expression throughout the entire apical dome of the SAM and procambium, including the incipient leaf primordium. (**l**) Expression throughout the entire apical dome of the SAM, procambium, and throughout the entire young leaf primordium, lacking from the LAI. (**m**) Expression in the apical region, procambium, and margins; expression is discrete on the adaxial margin. (**n**) Expression in the apical region where pinnae are developing (white arrows), procambium, and adaxial margin. St, leaf developmental stages following Figure 3; asterisks, SAM; brackets, LAI. Bars = 40 um, (except n = 80 um).

All leaves from the three *Elaphoglossum* species studied arise as lateral organs from the flank of the SAM. Leaf initiation (Stage 0) is detected by the enlargement of a superficial cell on the flank of the SAM. The morphology of leaf primordia of species with simple and divided leaves appears similar from Stages 0–2; all primordia are simple, and no outgrowths are detectable in both simple and divided leaved species (Figure 4 and Figure 5). All leaf primordia are characterized by the presence of an enlarged cell at the apex—the leaf apical initial (LAI), surrounded by small cells forming a wedge shape around it, together comprising the leaf apical meristem (LAM) (LAI clearly seen in Figure 4b,c,f,g,j and Figure 5c,g,k,l). In Stage 3 of leaf development, the apex of the leaf primordium apex is extremely curved towards the shoot apex (Figure 4c,l). In Stage 4 of development, in species with divided leaves, subdivisions are detectable in the apical portion of the leaf primordium, but the primordium and its pinnae are still coiled (Figure 5n).

### 2.3. Patterns of Cell Division in Simple and Divided Leaves of Elaphoglossum

Generally, the first approach to study leaf development is to look at leaf primordia at different stages of development under a scanning electron microscope (SEM). In our studied species, this approach was not feasible owing to the presence of scales, which develop early, are large, and are copious around the SAM and leaf primordia (Figure S2). Instead, to better understand the patterns of cell division in developing simple and divided leaves of the three closely related species of *Elaphoglossum* with diverse leaf morphologies, we used in situ hybridization analyses of the *H4* genes. Using degenerate primers, we recovered one copy of *H4* for each of the three studied species (*EllH4*, *EppH4*, *EpsH4*, Appendix B).

We found that *H4* is expressed in punctate patterns during Stage 1 (Figure 4e,j) and Stage 2 (Figure 4b,f,g,k). In these developmental stages, the *H4* expression pattern is similar in species with simple and divided leaves, suggesting that cell divisions in young leaf primordia are similar, occurring randomly throughout the primordium and infrequently in the LAI, but more frequently in the cells surrounding the LAI. In primordia of species with simple and divided leaves, *H4* expression in Stage 3 is detected in the apical region behind the LAI and in the procambium (Figure 4c,l). However, at Stage 3, in the species with simple leaves, *H4* expression along the margins is continuous (Figure 4c), while H4 expression in divided leaves is discontinuous in the abaxial side, being detected in discrete regions of the leaf margins (Figure 4l). Although *H4* expression is clearly different at Stage 3 between species with simple and divided leaves, morphologically, these primordia are indistinguishable (compare Figure 4c with Figure 4l). Transverse sections of Stage 4 leaf primordia of simple and divided developing leaves show little expression of *H4* in the petiole and random expression in the lamina (Figure 4h,m).

### 2.4. Class I KNOX Gene Expression Patterns in Simple and Divided Leaves of the Fern Genus Elaphoglossum

To better understand the molecular genetic basis underlying fern leaf morphological diversity and to compare our data with what is known for other ferns and seed plants, we used in situ hybridization to determine whether changes in gene expression correlate with changes in leaf morphology in the species with simple and divided leaves of our study group in the genus *Elaphoglossum* (Figure 1). We compared and analyzed the expression profiles of two of the fern copies of the meristem maintenance *Class I KNOX* genes (orthologous to all the well-known *Class I KNOX* angiosperm genes) specific to *Elaphoglossum lloense* (simple leaves, only *EllC1KNOX2* copy), *E. peltatum* f. *peltatum* (divided leaves, *EppC1KNOX1*, *EppC1KNOX2*), and *E. peltatum* f. *standleyi* (simple leaves, *EpsC1KNOX1*, *EpsC1KNOX2*) (Figure 2 arrow heads).

We found that, in our studied species of *Elaphoglossum*, patterns of expression of both *Class I KNOX* copies are similar to each other throughout leaf development (compare patterns of expression in Figure 5 with Figure S3). *Class I KNOX* genes are expressed throughout the entire apical dome of the shoot meristem and the procambium regardless of leaf morphology (Figure 5b,f,k,l). Interestingly, we found evidence that indicates *Class I KNOX* are downregulated in incipient leaf primordia in at least one of the species with simple leaves (Figure 5b). Because fern roots develop in the stem just beneath the leaf primordium [50], we also detected *Class I KNOX* expression at developing roots, likely at the root apical meristem (RAM) (Figure S3).

In *E. lloense* and *E. peltatum* f. *standleyi,* the species with simple leaves, *Class I KNOX* are expressed throughout the entire young leaf primordium (not including the LAI) at Stage 1 (Figure 5c,g). This expression is maintained throughout Stage 2 in the leaf apical region (including the LAI), procambium, and in the margins (Figure 5h). Later in development, in Stages 3 and 4, *Class I KNOX* expression is restricted to the leaf primordium apical region, procambium, and it starts disappearing or it is absent from the margins (Figure 5d,i).

In *E. peltatum* f. *peltatum,* the species with divided leaves, *Class I KNOX* are expressed throughout the entire young leaf primordium (not including the LAI) at Stage 1 (Figure 5k,l). This expression is maintained throughout Stage 2 in the leaf apical region (including the LAI), the procambium, and in a discontinuous pattern in the margins (Figure 5m). Later in development in Stage 4, when divisions are evident at the apical region in older leaf primordia of the species with divided leaves, *Class I KNOX* expression is detected at the apical region and developing divisions, in the procambium, and in the margins of the leaf primordium adaxially (Figure 5n).

## 3. Discussion

### 3.1. Evolutionary History of Class I KNOX Transcription Factors in Ferns

Our results showed that, within vascular plants, *Class I KNOX* lycophyte sequences are recovered in four different clades, successively sister to euphyllophytes, and not reciprocally monophyletic (Figure 2). The non-monophyly of lycophyte sequences might suggest ancient duplication events of the only inherited *Class I KNOX* gene in the ancestor of all vascular plants, or it might be the result of high rates of evolution combined with limited sequence data (see [51,52] for similar results in different gene phylogenies). Further analyses of additional lycophyte genomes are necessary to better understand the evolutionary history of *Class I KNOX* in lycophytes and their relationship with those of other vascular plants.

Our phylogenetic hypothesis recovered fern sequences as a monophyletic group sister to *Class I KNOX* genes of seed plants, which suggests that all fern sequences are putative orthologs to the one known *Class I KNOX* seed plant lineage. Although additional expression analyses using RNAseq techniques might reveal additional *Class I KNOX* copies in certain fern groups, we found ferns have at least three copies of *Class I KNOX* genes (Figure 2). The ferns *Class I KNOX* Copy3, which is recovered sister to the other two, was found exclusively in sequences of the water fern order Salviniales (sensu [53]). Two of those ferns, *Azolla filiculoides* and *Salvinia cucullata,* correspond to the family Salviniaceae and are the only fern species whose genomes are currently sequenced and publicly available [54]. The other sequence corresponds to *Marsilea minuta* in the family Marsiliaceae and was revealed during our mining for *KNOX* genes using degenerate primers. Salviniaceae and Marsiliaceae, which are sister families, not only predominantly grow in water, but also are the only ferns that are heterosporic [53]. Although further genome sequencing may reveal additional fern taxa that have the *Class I KNOX* Copy3, it is also possible that this copy may be restricted to heterosporic ferns and play a role in heterospory.

The other two copies of fern *Class I KNOX* found, Copy1 and Copy2, are sister to each other and show phylogenetic relationships largely consistent with recently published fern species phylogenies [53,55,56], suggesting the two copies diversified during the evolution of ferns (Figure 2). Obtaining representative fern genomes and conducting further comparative analyses of the evolutionary history of different gene families will be important to determine if the duplication that led to Copies 1 and 2 of *Class I KNOX* genes in ferns was the result of the whole-genome duplication predating the core Leptosporangiate ferns inferred by Li et al. [54] or of another mechanism of gene duplication.

### 3.2. Class I KNOX Genes Are Expressed in Shoot and Leaf Fern Meristems

Developmentally, both seed plant and fern leaves (megaphylls) arise as lateral organs from the flank of an indeterminate SAM in a distinct phyllotaxy, have adaxial/abaxial identities, and are determinate organs. Fern leaves, however, differ from seed plant leaves in several aspects. Morphological and anatomical studies have shown that, in general, development of the fern leaf is from the leaf apical meristem (LAM) and the marginal meristem (MM). The LAM is composed by a leaf apical initial (LAI) and its derivatives, the LAI is an enlarged cell located at the tip of the fern leaf primordium [57,58,59]. The MM is located at the periphery of developing leaf primordia and is composed of marginal and submarginal initials and has been argued to be organized similar to a SAM [49,57,60,61,62,63]. In ferns, the MM makes a major contribution to lamina formation, and remains active until the general morphology of the leaf is established and the location of all procambium has been determined [47,62,64].

Class I KNOX proteins in angiosperms have been shown to be generally necessary for meristem maintenance [14,15,16,17,18,19,20,21]. We found *Class I KNOX* expression in the SAM, LAM, and RAM of ferns with simple and divided leaves (Figure 5 and Figure S3). We also found *Class I KNOX* expression in the margins in the early development of simple and divided leaved *Elaphoglossum* species (Figure 5). The *Class I KNOX* expression in the margins of fern leaf primordia reflects the persistent meristematic activity of the MM and the interpretation of the fern leaf margin as a region of sustained meristematic activity [47,49,63].

### 3.3. Class I KNOX Gene Expression in Fern Leaves Recapitulates Shoot Expression

Compared with angiosperms, fern leaves have longer meristematic activity and maturation toward the apex [65,66]. This has been explained by the presence in fern leaf primordia of the LAM (LAI and derivatives) [57,58,59]. Angiosperm leaves do not have apical initials and, contrary to ferns, leaf growth is not limited to the apex and margins; instead, it can be diffuse with meristematic activity throughout the developing leaf, with some angiosperms having an intercalary meristem and plate meristem that give rise to most cells of the lamina [45].

Anatomical and experimental studies have demonstrated that fern leaf primordia have shoot-like characteristics, transitioning later to determinate fate when compared with angiosperms [65,66,67,68,69,70,71,72]. The persistent *Class I KNOX* expression we found at the LAM (LAI and surrounding cells) of developing leaves in the three species of *Elaphoglossum* supports these anatomical and experimental studies and agrees with previous findings of other comparative genetic studies [9,73,74]. Studies of angiosperm species with divided leaves, such as tomato, have considered divided leaves more shoot like, and this was reflected by persistent *Class I KNOX* expression in the leaves [25].

*Class I KNOX* genes are expressed in the SAM of ferns [9,32,33]. Our previous study, concentrated on *Class I KNOX* expression in the shoot apical fern meristem, found expression throughout the shoot apical dome (apical initial and surrounding cells) and reported that, in 40% of the experiments, expression of *Class I KNOX* was not detected in the shoot apical initial [33]. We found that this pattern of expression is recapitulated in the LAM of both simple and divided leaves of ferns, with expression detected at the apical portion of the leaf primordium throughout development but captured in the LAI intermittently (Figure 5). The recapitulated *Class I KNOX* expression in the SAM and in the LAM of leaf primordia of both simple and divided leaves in ferns, suggest that a similar developmental mechanism is present during development in fern shoots and fern leaves, giving further genetic and molecular support for the shoot-like nature (persistent meristematic activity) of fern leaves compared with seed plants leaves. Our findings support the partial shoot theory of leaf evolution proposed by Arber [75,76], who considered the shoot to be the fundamental organ of the plant, and that all leaves were partial shoots because their indeterminate growth and radial symmetry are repressed.

### 3.4. Development of Simple and Divided Leaves in Ferns

Anatomical and morphological studies of fern leaf development have shown that primary fern leaf primordium development is owing to the growth and divisions of the LAM and the MM [57,60,61,63]. The patterns of *H4* expression we found in simple and divided leaves support these findings and suggest that, regardless of final morphology, cell divisions in early developing leaf primordia are similar in ferns (Figure 4).

The expression patterns of *Class I KNOXs* we found at Stages 0–2 of leaf development are also similar in the three species of *Elaphoglossum,* suggesting that *Class I KNOX* expression is necessary for leaf development, but that early *Class I KNOX* expression cannot explain the morphological differences between simple and divided leaves in ferns (Figure 5). Only later in leaf development (Stages 3 and 4) does *Class I KNOX* expression and cellular division patterns (*H4* expression) change between simple and divided leaves (Figure 4 and Figure 5). Notably, at Stages 3 and 4, the expression of *Class I KNOX* persisted at the margins of leaf primordia of species with divided leaves (even after divisions develop) compared with species with simple leaves (Figure 5i,n). This persistent expression in the species with divided leaves compared with simple leaves indicates that temporal and spatial patterns of expression of *Class I KNOX* genes correlate with different fern leaf morphologies. This suggests that, although Class I KNOX alone is not sufficient to promote divided leaf development in ferns, *Class I KNOX* genes might contribute to the morphological variation between simple and divided leaves in ferns, as has been shown for angiosperms [22].

Previous anatomical and ontogenetic studies have suggested that, in leaf primordia of ferns with divided leaves, the LAM (LAI and derivatives) remains active and divisions occurred when the MM becomes interrupted in a regular manner, and some regions lose their meristematic potential [47,57,60,61,63,64,73]. The *Class I KNOX* expression in the margins of leaf primordia of ferns with simple and divided leaves highlights the critical role marginal meristems play in leaf development in ferns. Interestingly, both of our studied species with simple leaves have similar *Class I KNOX* expression patterns, even though the simple leaved *E. peltatum* f. *standleyi* is only a different form of the same species as the divided leaved *E. peltatum* f. *peltatum* [35].

Notably, we detected down-regulation of *Class I KNOX* in incipient leaf primordia in one of the *Elaphoglossum* species with simple leaves (Figure 5b), but not in the divided leaved species (Figure 5k and Figure S3c). Although a lack of downregulation at Stage 0 in fern species with divided leaves has also been reported before [9], it could be that this precise developmental stage is difficult to capture in fern leaf development or that there is a difference in downregulation of *Class I KNOX* between species of ferns with simple and divided leaves.

### 3.5. A Conserved Mechanism of Leaf Dissection in Megaphylls

*Class I KNOX* expression and function in leaf primordia of angiosperms has been shown to underlie leaf morphology differences in angiosperms [9,22,77]. In angiosperms with simple leaves, *Class I KNOX* are only expressed in the SAM, and down-regulated in incipient leaf primordia and in mature leaves [14,17,20]. Whereas, in most angiosperms with divided leaves, *Class I KNOX* are expressed in the SAM, down-regulated in incipient leaf primordia, expressed throughout the young leaf primordia, and expressed in sites of leaflet initiation in older leaf primordia [9,22]. A notable exception is the divided leaved species of tomato, where down-regulation of *Class I KNOX* in P0 has not been found [24,25].

We found a fundamental difference in leaf development in the *Class I KNOX* expression patterns between ferns and seed plants with simple leaves. In ferns with simple leaves, contrary to what has been reported for most angiosperms, *Class I KNOX* are expressed early in leaf development and maintained in the apical region of the developing leaf (Figure 5). On the other hand, similar to angiosperms with divided leaves, we found that, in ferns with divided leaves, *Class I KNOX* are expressed throughout the young leaf primordium and that this expression persists at the margins of leaf primordia (Figure 5). Moreover, in ferns with divided leaves, *Class I KNOX* expression persists in the apical region of the leaf primordium until very late in development. It has been shown that *Class I KNOX* expression in leaves was independently recruited to control divided leaf development in multiple seed plant lineages [9]. Our *Class I KNOX* expression data in a fern with divided leaves suggest that this genetic mechanism might have also been independently recruited in ferns to control divided leaf development.

In angiosperms, there are several proteins and hormones known to act in the Class I KNOX pathway that affect leaf shape, including the proteins belonging to the NO APICAL MERISTEM/CUP-SHAPED COTYLEDON (NAM/CUC) and ASYMMETRIC LEAVES/ROUGH SHEATH2/PHANTASTICA (ARP) families of transcription factors, that are known to be redeployed to make leaflets in a divided leaf [29,78,79,80,81]. The complex patterns of *Class I KNOX* expression we found in all our studied fern species, and the persistent *Class I KNOX* expression in leaf margins and divisions of species with divided leaves compared with the species with simple leaves, could also be mediated by auxin maxima that are generated by PIN1, an auxin efflux transporter [22,29,82,83], as well as changes in protein partners such as members of the *NAM/CUC* family of transcription factors that maintain *Class I KNOX* expression in a positive feedback loop in the SAM and within divided leaves for leaflet formation [77,79,84]. Phylogenetic, expression, and functional studies of these genes in all the major lineages of vascular plants will be important to fully understand to what extent the developmental genetic network underlying megaphyll morphological diversity is conserved in Euphyllophytes (ferns and seed plants).

### 3.6. Class I KNOX Genes and Megaphyll Evolution

The homology of megaphylls is still highly debated, and even within the ferns, it is not clear if leaves are homologous [5,7]. Previous comparative studies have come to different conclusions about the conservation of a leaf developmental network between ferns and seed plants [9,10,52,85,86]. Conservation in a leaf developmental program across ferns and seed plants was suggested by comparative expression studies of two leaf developmental genes, *Class I KNOX* and *Class III HD-Zip* [10,52]. The *Class I KNOX* downregulation in leaf primordia of fern species with simple leaves we reported here, along with the other similarities in *Class I KNOX* expression between angiosperms and ferns (SAM and margins of leaf primordia in species with divided leaves, Figure 5), supports the hypothesis of a conservation in a leaf developmental program across ferns and seed plants, suggesting an independent co-option of a common ancestral mechanism for leaf development.

Overexpression and complementation studies in angiosperms suggest that *Class I KNOX* homologs from ferns can provide some of the same functions as endogenous angiosperm genes [10,32]. However, a recent comparative study across ferns showed differential expression of another leaf transcription factor, *Class III HD-Zip* in the SAM of ferns, where expression was not detected in *Equisetum* and *Osmunda,* but was detected in leptopsorangiate ferns [52]. Additional expression studies in diverse fern species as well as knockouts will be necessary to better understand what these genes do in their native context, to further test hypotheses of leaf evolution, and to better understand the differences in connection with the leaf developmental network across ferns.

## 4. Materials and Methods

### 4.1. Sampling for the Phylogenetic Analyses of Class I KNOX Genes

To gain a more detailed evolutionary history of *Class I KNOX* in ferns and to discover the putative *Elaphoglossum Class I KNOX* gene copies for our expression studies, we obtained representative species across the fern and lycophyte phylogeny from publicly available databases and by cloning. We included *Class I KNOX* genes previously published from the lycophytes *Selaginella krausiana* [10], *Huperzia selago* and *Isoetes tegetiformans* [87], and *Lycopodium deuterodensum* [88]; from the ferns *Ceratopteris richardii* [32], *Elaphoglossum peltatum* f. *peltatum* [33], and *Equisetum diffusum* [88]. We got these sequences from GenBank, the 1KP plant transcriptome project (http://www.onekp.com, accessed May 2018) databases, or directly from the published papers. BLAST similarity searches (Altschul et al., 1990) in the lycophyte *Selaginella moellendorffii* genome available in Phytozome (https://phytozome.jgi.doe.gov, last accessed May 2018) were used to identify *Class I KNOX* copies in *S. moellendorffii*. BLAST searches were also conducted in the fern genomes of *Azolla filiculoides* and *Salvinia cucullata,* available in Fernbase (https://www.fernbase.org/, last accessed May 2019). Further lycophyte and fern sequences were obtained using degenerate primers previously published [33]. For the phylogenetic analyses, published sequences from GenBank for the other lineages of vascular plants (gymnosperms and angiosperms) were also included. *Class I KNOX Physcomitrium patens* sequences available at GenBank were used as outgroups and to root the trees. A list of all sampled species, provenance, and accession numbers is provided in Appendix A (these will be provided during review).

### 4.2. Sequence Analysis, Alignment, and Phylogenetic Analysis

New sequence contigs were assembled using Geneious V. 11 (Biomatters Ltd., New Zealand). Sequences were compiled and cleaned to keep just the open reading frame. Nucleotide sequences were aligned using the online version of MAFFT v.7 [89]. The alignment was refined by hand, using Mesquite V. 3.5 [90], considering protein domains and amino acid motifs that have been reported as conserved for *KNOX* genes. A matrix that included KNOX, the ELK, and the TALE-HD was used for phylogenetic analyses. Phylogenetic relationships were inferred from the nucleotide data using Bayesian inference (BI). Analyses were performed on CIPRES (http://www.phylo.org) [91]. The best partition scheme was found with PartitionFinder2 [92], for the nucleotides matrix 15 data blocks were defined by dividing the matrix into five regions (KNOX 1 (first KNOX domain), KNOX 1–KNOX 2 (region between KNOX 1 and KNOX 2), KNOX 2 (second KNOX domain), KNOX 2–HD (region between KNOX 2 and the HD), and HD (ELK and TALE-HD domains)), and by dividing each region by codon position. Analyses were performed with nine subsets as estimated by the corrected Akaike Information Criterion (AICc) implemented in PartitionFinder2 (Table S1; see Supplemental Data with this article). For the amino acids matrix, the JTT+I+G model was used as estimated by the corrected Akaike Information Criterion (AICc) implemented in PartitionFinder2 [93]. For both matrices, BI analyses were conducted using MrBAYES 3.2.6 [93]. Two independent runs of 10 million generations were completed, with four chains each (three heated, one cold), using a chain temperature of 0.2 and uniform priors. Trees and parameters were sampled every 1000th generation. Samples corresponding to the initial phase of the Markov chains (25%) were discarded as burn-in. The applicability of this burn-in value was determined by the inspection of the likelihood scores and effective sample sizes. Post-burn-in trees were combined to obtain a single majority rule consensus tree and the respective posterior probabilities (PPs) of nodes. Trees were depicted using FigTree v1.4.3 (http://tree.bio.ed.ac.uk/software/figtree/).

### 4.3. Taxonomic Sampling for Gene Expression Studies

To better understand the molecular genetic basis for the differences in leaf form in ferns, we studied gene expression patterns of *Histone H4* genes (used as a cell division marker) and of *Class I KNOX* genes in developing leaf primordia of two taxa with simple leaves (*E. lloense* and *E. peltatum* f. *standleyi*) and one taxon with divided leaves (*E. peltatum* f. *peltatum*) belonging to the fern genus *Elaphoglossum* (Figure 1b–d). Additionally, we compared our results of *Class I KNOX* expression to what is known from similar studies performed in seed plants and lycophytes, in order to better understand what these leaf developmental genes tell us about megaphyll leaf evolution. 

For the expression analyses, the material of *E. lloense* was collected in the field in Ecuador (Vasco 865, *NY*), and both *E. peltatum* forms were sourced from specialist fern growers and kept in the Nolen glasshouses at the New York Botanical Garden (NYBG).

### 4.4. RNA and DNA Extraction and cDNA Synthesis

For RNA and DNA extraction, we preserved the material collected in the field in Ecuador in RNA*later* (Life Technologies, Carlsbad, CA, USA); for the living plants growing in the NYBG greenhouses, we preserved the material in liquid nitrogen. Total RNA was extracted from sporophyte shoot apices (including the SAM and young leaves), as previously described [94] with some modifications as follows. Approximately 5 g of tissue was ground to a fine powder in liquid nitrogen with a mortar and pestle. The powder was added to 25 mL of lysis buffer containing 0.1 M NaCl, 50 mM TrisHCl (pH 7.4), 50 mM EDTA (pH 8), 2% SDS, and proteinase K (200 ug/mL), and stirred at room temperature for 10–15 min. Cell debris was centrifuged at 10,000 rpm for 5 min, and the supernatant was extracted twice with an equal volume of phenol/chloroform/isoamylalcohol (50:48:2) and once with chloroform/isoamylalcohol (96:4), centrifuging each time at 10,000 rpm for 10 min. A volume of 0.1 of 3M NaOAc and 2.5 volumes Ethanol (ETOH) were added to the aqueous phase and centrifuged at 10,000 rpm for 5 min. The pellet was air dried from 5 min and resuspended in 700 uL DEPC-water. Then, 700 uL of LiCl (4M) was added and incubated overnight at 4 °C. The sample was centrifuged at 10,000 rpm for 10 min at 4 °C, the supernatant was discarded, and the pellet was resuspended in 200 uL DEPC water. Then, 20 uL of NaOAc (3M) and 500 uL ETOH were added and left at −20 °C for 30 min. The sample was centrifuged at 10,000 rpm for 10 min at 4 °C, and then washed with 70% ETOH made with DEPC water. Finally, the pellet was air dried and resuspended in 20 uL of DEPC water. Samples collected in RNA*later* were extracted with the same protocol, but the tissue was ground in the lysis buffer. cDNA was synthesized using Superscipt III (Invitrogen, Carlsbad, CA, USA) according to the manufacturer’s instructions.

### 4.5. Primer Design and PCR

*Histone H4* and *Class I KNOX* sequences of the three *Elaphoglossum* species were isolated by PCR with degenerate primers (01H4f5’ATGTCWGGMMGRGGWAAGGGAGG, 01H4r5’ CCRAADCCRTARAGVGTHCKKCC, 01KNOXf5’ CCBGARCTBGACMABTTYATGG, and 02KN OXr5’ CCAGTGSCKYTTCCKYTGRTTDATRAACC) and by 5’ RACE (Clontech Laboratories Inc., Mountain View, CA, USA) according to the manufacturer’s protocol. PCR reactions used cDNA as template and forward and reverse degenerate primers. PCR products were cleaned and cloned directly into the pCRII vector (TOPO TA cloning kit, Invitrogen, Carlsbad, CA, USA). A total of 20–30 colonies were grown in LB culture and plasmid DNA was isolated. Clones representing different banding patterns were sequenced by the Sanger method (Macrogen, USA) and BLAST was used to compare sequences in NCBI.

### 4.6. In Situ Hybridization

Tissues were fixed in the field for *E. lloense* or at NYBG for both *E. peltatum* forms in formaldehyde acetic acid for 2–4 h, and then dehydrated through a graded ethanol series to 100% ethanol. Tissue was embedded in Paraplast X-tra (Fisher brand) and sectioned on a microtome to 10 um. Sections were placed on ProbeOn Plus slides (Fisherbrand, Pittsburgh, PA). Gene-specific fragments for all the recovered *Elaphoglossum H4* and *Class I KNOX* copies (see results) were amplified using primers designed for this study (Figure S1 and Table S2). Digoxigenin labeled gene-specific probes were generated according to the manufacturer’s instructions (Roche Applied Science, Indianapolis, IN, USA). Slides were left on a hot plate at 42 °C overnight. Treatment of cells and tissues prior to hybridization was performed as previously described [95]. Hybridizations, washes, blocking, antibody incubation, and detection were performed as in Torres et al. [96], except hybridization was performed overnight in 50% formamide humidified box at 55 °C. Sense probes were used as negative controls on pairs of slides and run in parallel with antisense probes. Sense probes gave no staining to illustrate that none of the tissue was sticky, as already indicated by different expression patterns exhibited by antisense *KNOX* and *H4*. Slides were examined and photographed on a Zeiss Axioskop microscope equipped with a Zeiss Axiocam digital camera.

## Supplementary Materials

Supplementary Materials can be found at https://www.mdpi.com/1422-0067/21/15/5180/s1. Table S1. Best partition scheme and models for the aligned *Class I KNOX* matrix as estimated by the corrected Akaike Information Criterion (AICc) implemented in PartitionFinder2. Table S2. Forward and reverse primers designed for in-situ hybridizations. Figure S1. (a) Nucleotide and (b) amino acid alignment of the three copies of *Class I KNOX* genes recovered in ferns. In the nucleotide alignment, dark and white bars show the location of the forward and reverse primers respectively, designed for in-situ hybridizations. Figure S2. Scanning electron microscope images of shoot apices of *Elaphoglossum peltatum* f. *peltatum* showing massive presence of scales over the SAM and coiled young leaf primordia. (a) Shoot apex completely covered by scales. (b) Stage 3, leaf primordium completely covered by scales. (c) Stage 4 leaf primordium with coiled subdivisions (pinnae) completely covered by scales. (d) Late Stage 4 leaf primordium, only at this stage of development is lamina visible. Star indicates the putative location of the SAM; L, leaf primordium; P, pinna; scales are highlighted with dotted lines. Figure S3. Additional expression of *Class I KNOX* genes during leaf development in species of the fern genus *Elaphoglossum* with simple and divided leaves. (a,b) *Elaphoglossum peltatum* f. *standleyi* (simple leaves), expression of *EpsC1KNOX1,* longitudinal sections through the SAM, and/or leaf primordia. (a) Expression throughout the entire apical dome of the SAM and procambium, including expression in the incipient leaf primordium (Stage 0). (b) Expression throughout the entire young leaf primordium including the leaf apical initial (LAI); expression in the root apical meristem (RAM). (c–g) *Elaphoglossum peltatum* f. *peltatum* (divided leaves). (c–f) Expression patterns of *EppC1KNOX1,* longitudinal sections through the SAM, and/or leaf primordia. (c) Expression throughout the entire apical dome of the SAM and procambium, including expression in the incipient leaf primordium (Stage 0). (d) Expression throughout the entire young leaf primordium including the LAI. (e) Expression throughout the entire apical dome of the SAM and procambium; expression in the root apical meristem (RAM). (f) Expression throughout the entire apical dome of the SAM and procambium, including expression in the incipient leaf primordium; expression in the apical region of an older leaf primordium (right). (e) Expression of *EppC2KNOX1* in the leaf primordium, procambium and root primordium. Black arrowheads, leaf primordia; brackets, LAI; stars, SAM; white arrows = root primordium; white arrowheads, procambium; bars = 40 um.

## Abbreviations

C1KNOX	*Class I KNOTTED1-like HOMEOBOX*
LAI	Leaf Apical Initial
LAM	Leaf Apical Meristem
MM	Marginal Meristem
RAM	Root Apical Meristem
SAM	Shoot Apical Meristem

## Appendix A

*Class I KNOX* sequences used in this study. The information is presented in the following order: **Lineage**, ***species***, name of the sequence in the tree of Figure 2, database, accession number. The first instance of a lineage and species is given in bold. Sequences MT680030- MT680042 are new sequences generated for this study.

**Bryophytes: *Physcomitrium patens*** (Hedw.) Bruch & Schimp., Physcomitrella_PpMKN2, NCBI, XM_001758540. Physcomitrella_PpMKN4, NCBI, XM_001781425. Physcomitrella_PpMKN5, NCBI, XM_001778213. **Lycophytes: *Huperzia selago*** (L.) Bernh. ex Schrank & Mart., Huperzia_HsKNOX1_1, NCBI, KX761181. Huperzia_HsKNOX1_2, NCBI, KX761182. ***Huperzia squarrosa*** (G. Forst.) Trevis., Huperzia_HsqC1KNOX1, NCBI, MT680030. ***Isoetes riparia*** Engelm. ex A. Braun, Isoetes_IrC1KNOX1, NCBI, MT680031. Isoetes_IrC1KNOX2, NCBI, MT680032. ***Isoetes tegetiformans*** Rury, Isoetes_ItKNOX1, 1KP, PKOX_2098898. ***Lycopodium deuterodensum*** Herter, Lycopodium_LdC1KNOX1, 1KP, PQTO-2010435. Lycopodium_LdC1KNOX2, 1KP, PQTO-2081329. ***Selaginella kraussiana*** (Kunze) A. Braun, Selaginella_SkKNOX1, NCBI, AY667449. Selaginella_SkKNOX2, NCBI, AY667450. ***Selaginella moellendorffii*** Hieron., Selaginella_SmKNOX1, NCBI, XM_002988279. Selaginella_SmKNOX2, NCBI, XM_002977393. **Gymnosperms, *Picea abies*** (L.) H. Karst., Picea_PaHBK1, NCBI, AF063248. Picea_PaHBK2, NCBI, AF483277. Picea_PaHBK3, NCBI, AF483278. Picea_PaHBK4, NCBI, DQ257981 & DQ258006. ***Pinus taeda*** L., Pinus_PtKN1, NCBI, AY680402. Pinus_PtKN2, NCBI, AY680403. Pinus_PtKN3, NCBI, AY680404. Pinus_PtKN4, NCBI, AY680387 & AY680398. **Angiosperms, *Arabidopsis thaliana*** (L.) Heynh., Arabidopsis_AtSTM, NCBI, NM_104916. Arabidopsis_AtKNAT1/BP, NCBI, U14174.1. Arabidopsis_AtKNAT2, NCBI, NM_105719. Arabidopsis_AtKNAT6, NCBI, NM_102187. ***Zea mays*** L., Zea_ZmKn-1, NCBI, X61308. Zea_ZmrRS1, NCBI, L44133. Zea_Zmlg3, NCBI, NM_001112038. Zea_ZmKNOX5/lg4b, NCBI, NM_001111615.2. **Ferns, *Angiopteris evecta*** (G. Forst.) Hoffm., Angiopteris_AeC1KNOX1, NCBI, MT680033. ***Azolla filiculoides*** Lam., Azolla_AfC1KNOX1A, FernBase, Azfi_s2491.g111832 and Azfi_s0350.g066570. Azolla_AfC1KNOX1B, FernBase, Azfi_s0006.g009595. Azolla_AfC1KNOX3, FernBase, Azfi_s2342.g110698 and Azfi_s0350.g066569. ***Ceratopteris richardii*** Brongn., Ceratopteris_CrKNOX1, NCBI, AB043954. Ceratopteris_CrKNOX2, NCBI, AB043956. ***Cyathea dregei*** Kunze, Cyathea_CdC1KNOX2, NCBI, MT680034. ***Elaphoglossum ciliatum*** (C. Presl) T. Moore, Elaphoglossum_EcC1KNOX2, NCBI, MT680035. ***Elaphoglossum lloense*** (Hook.) T. Moore, Elaphoglossum_EllC1KNOX2, NCBI, MT680036. ***Elaphoglossum peltatum f. peltatum*** (Sw.) Urb., Elaphoglossum_EppC1KNOX1, NCBI, KT382287. Elaphoglossum_EppC1KNOX2, NCBI, KT382288. ***Elaphoglossum peltatum f. standleyi*** (Maxon) Mickel, Elaphoglossum_EpsC1KNOX2, NCBI, MT680037. Elaphoglossum_EpsC1KNOX1, NCBI, MT680038. ***Elaphoglossum tripartitum*** (Hook. & Grev.) Mickel, Elaphoglossum_EtC1KNOX2, NCBI, MT680039. ***Equisetum diffusum*** D. Don, Equisetum_EdKNOX1A, 1kp, CAPN-2006400. Equisetum_EdKNOX1B, 1kp, CAPN-2006401. ***Marsilea minuta*** L., Marsilea_MmC1KNOX2, NCBI, MT680040. Marsilea_MmC1KNOX3, NCBI, MT680041. ***Pilularia globulifera*** L., Pilularia_PgC1NOX2, NCBI, MT680042. ***Salvinia cucullata*** Roxb., Salvinia_ScC1KNOX1A, FernBase, Sacu_v1.1_s0115.g020964. Salvinia_ScC1KNOX1B, FernBase, Sacu_v1.1_s0011.g005308. Salvinia_ScC1KNOX3, FernBase, Sacu_v1.1_s0091.g018785.

## Appendix B

*Histone H4* sequences recovered in this study. The information is presented in the following order: ***species***, name of the sequence, database, accession number. The first instance of a lineage and species is given in bold. All sequences are newly generated for this study.

***Elaphoglossum lloense*** (Hook.) T. Moore, EllH4, NCBI, MT776685. ***Elaphoglossum peltatum* f. *peltatum*** (Sw.) Urb., EppH4, NCBI, MT776686. ***Elaphoglossum peltatum* f. *standleyi*** (Maxon) Mickel, EpsH4, NCBI, MT776687.
