# Supplementary material for: Simple and Divided Leaves in Ferns: Exploring the Genetic Basis for Leaf Morphology Differences in the Genus Elaphoglossum (Dryopteridaceae)
Source: Int J Mol Sci. 2020 Jul 22;21(15):5180. doi: 10.3390/ijms21155180 (PMC7432805; doi:10.3390/ijms21155180)

Vasco and Ambrose—International Journal of Molecular Sciences– Figure S2

**Figure S2.** Scanning electron microscope images of shoot apices of *Elaphoglossum peltatum* f. *peltatum* showing massive presence of scales over the SAM and coiled young leaf primordia. (a) Shoot apex completely covered by scales. (b) Stage 3, leaf primordium completely covered by scales. (c) Stage 4 leaf primordium with coiled subdivisions (pinnae) completely covered by scales. (d) Late Stage 4 leaf primordium, only at this stage of development lamina is visible. Star indicates the putative location of the SAM, L, leaf primordium; P, pinna; scales are highlighted with dotted lines.


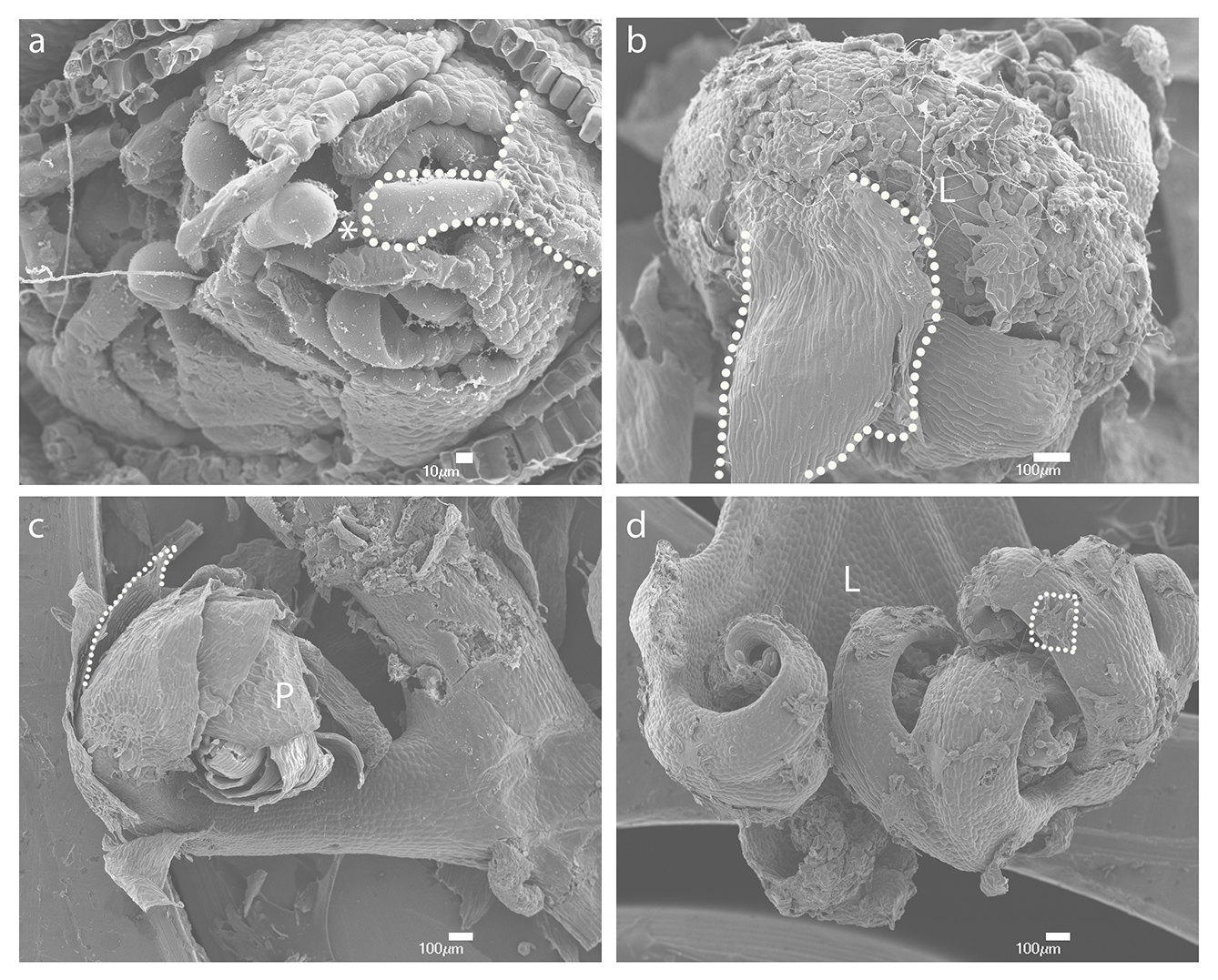

Supplement: Supplementary file 1 [file ijms-21-05180-s001.zip › Vasco&Ambrose_FigureS2_June26.docx]
