# Supplementary material for: Simple and Divided Leaves in Ferns: Exploring the Genetic Basis for Leaf Morphology Differences in the Genus Elaphoglossum (Dryopteridaceae)
Source: Int J Mol Sci. 2020 Jul 22;21(15):5180. doi: 10.3390/ijms21155180 (PMC7432805; doi:10.3390/ijms21155180)
Supplement: Supplementary file 1 [file ijms-21-05180-s001.zip › Vasco&Ambrose_TableS1.docx]

Vasco and Ambrose—International Journal of Molecular Sciences– Table S1

Table S1. Best partition scheme and models for the aligned *Class I KNOX* matrix as estimated by the corrected Akaike Information Criterion (AICc) implemented in PartitionFinder2.

| Subset | Best Model | # sites | Partition names |
| --- | --- | --- | --- |
| 1 | GTR+I+G | 82 | KNOX1_pos1, KNOX2_pos1 |
| 2 | GTR+I+G | 168 | KNOX1_pos2, KNOX2_pos2, HD_pos2 |
| 3 | GTR+I+G | 168 | KNOX1_pos3, KNOX2_pos3, HD_pos3 |
| 4 | HKY | 29 | KNOX1-KNOX2_pos1 |
| 5 | GTR+G | 144 | KNOX1-KNOX2_pos2, KNOX2-HD_pos2 |
| 6 | K80 | 29 | KNOX1-KNOX2_pos3 |
| 7 | GTR+G | 115 | KNOX2-HD_pos1 |
| 8 | HKY | 115 | KNOX2-HD_pos3 |
| 9 | GTR+I+G | 86 | HD_pos1 |
