# Supplementary material for: Simple and Divided Leaves in Ferns: Exploring the Genetic Basis for Leaf Morphology Differences in the Genus Elaphoglossum (Dryopteridaceae)
Source: Int J Mol Sci. 2020 Jul 22;21(15):5180. doi: 10.3390/ijms21155180 (PMC7432805; doi:10.3390/ijms21155180)
Supplement: Supplementary file 1 [file ijms-21-05180-s001.zip › Vasco&Ambrose_FigureS1_June26.docx]

Vasco and Ambrose—International Journal of Molecular Sciences– Figure S1

Figure S1. (a) Nucleotide and (b) amino acid alignment of the 3 copies of *Class I KNOX* genes recovered in ferns. In the nucleotide alignment, pink and black squares show the location of the forward and reverse primers respectively, designed for in-situ hybridizations.

**a.**

**
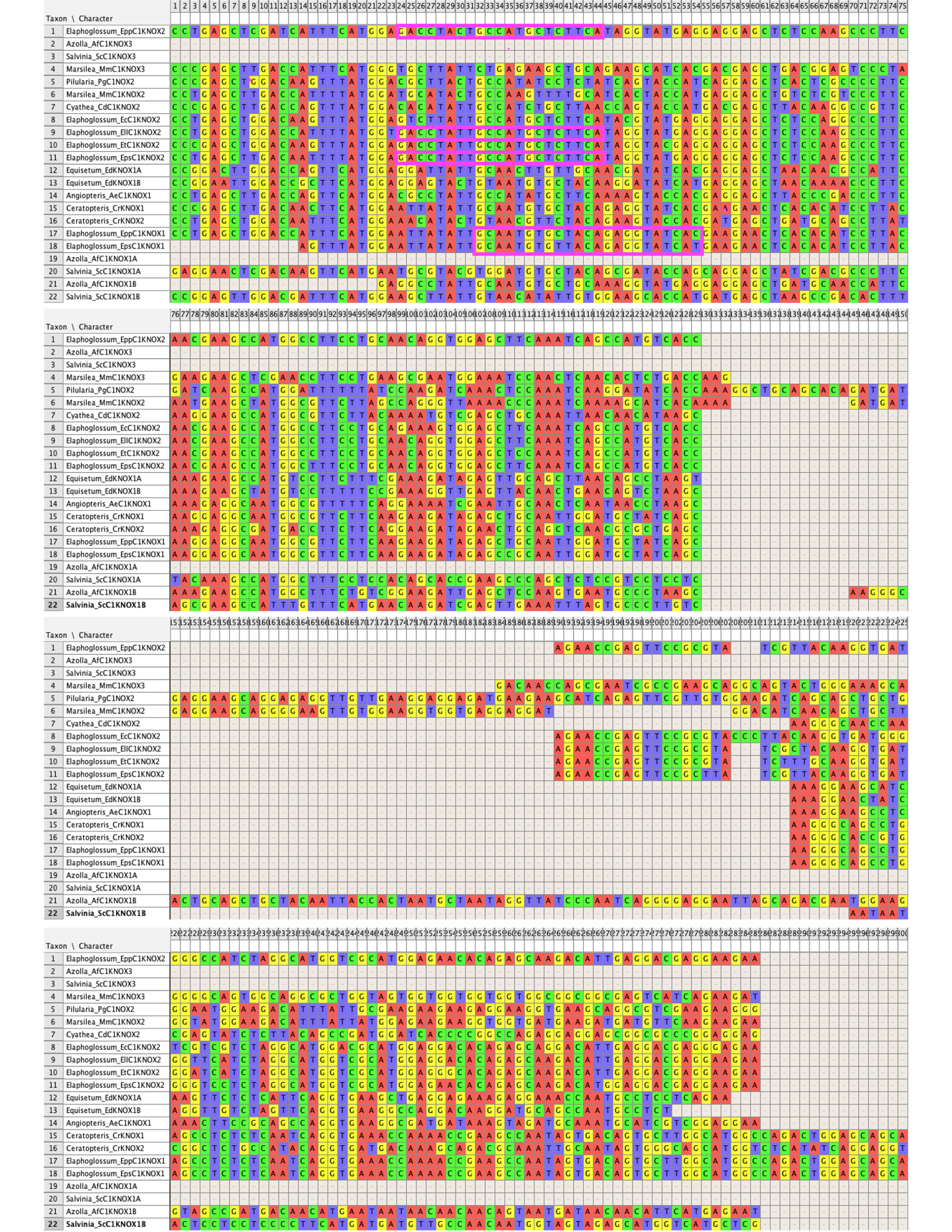
**


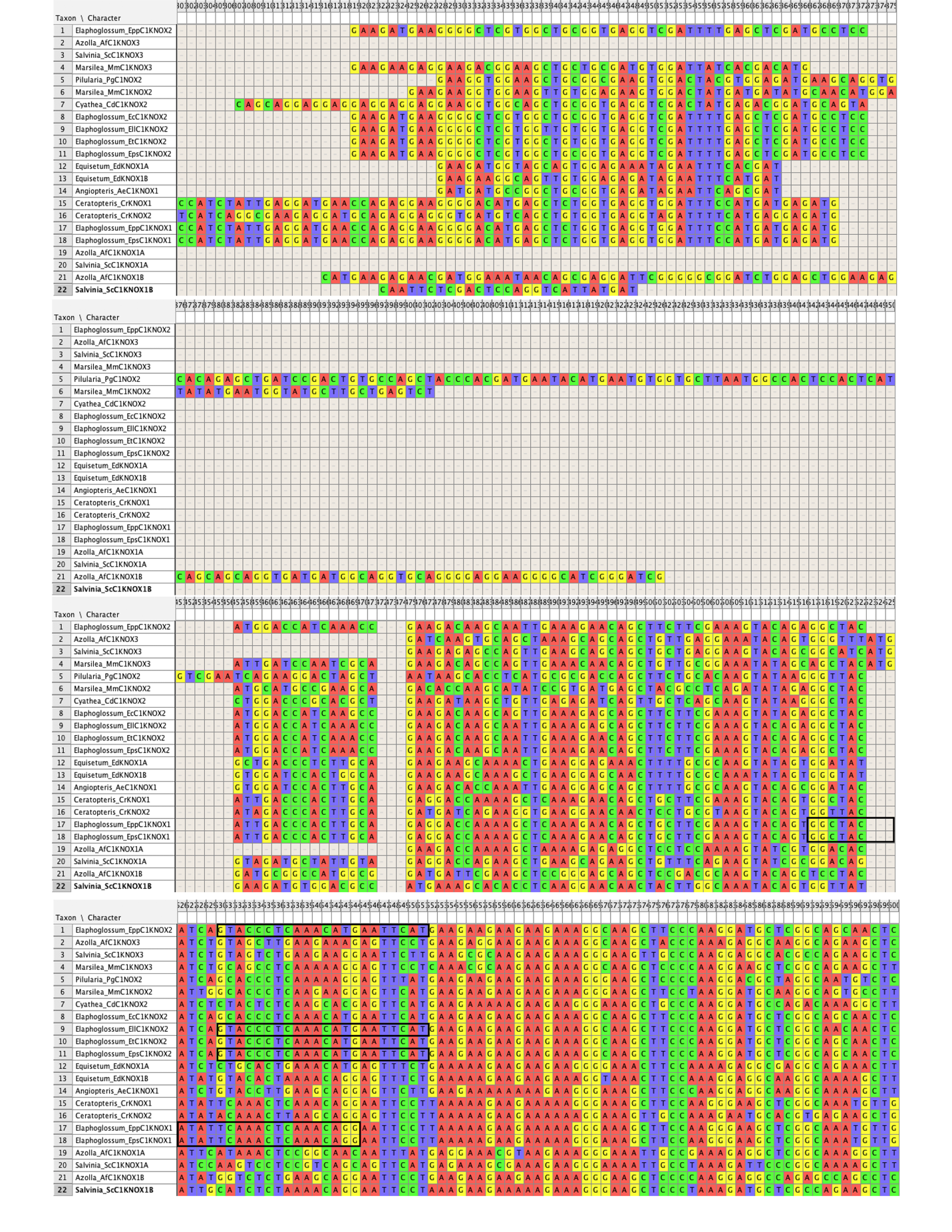


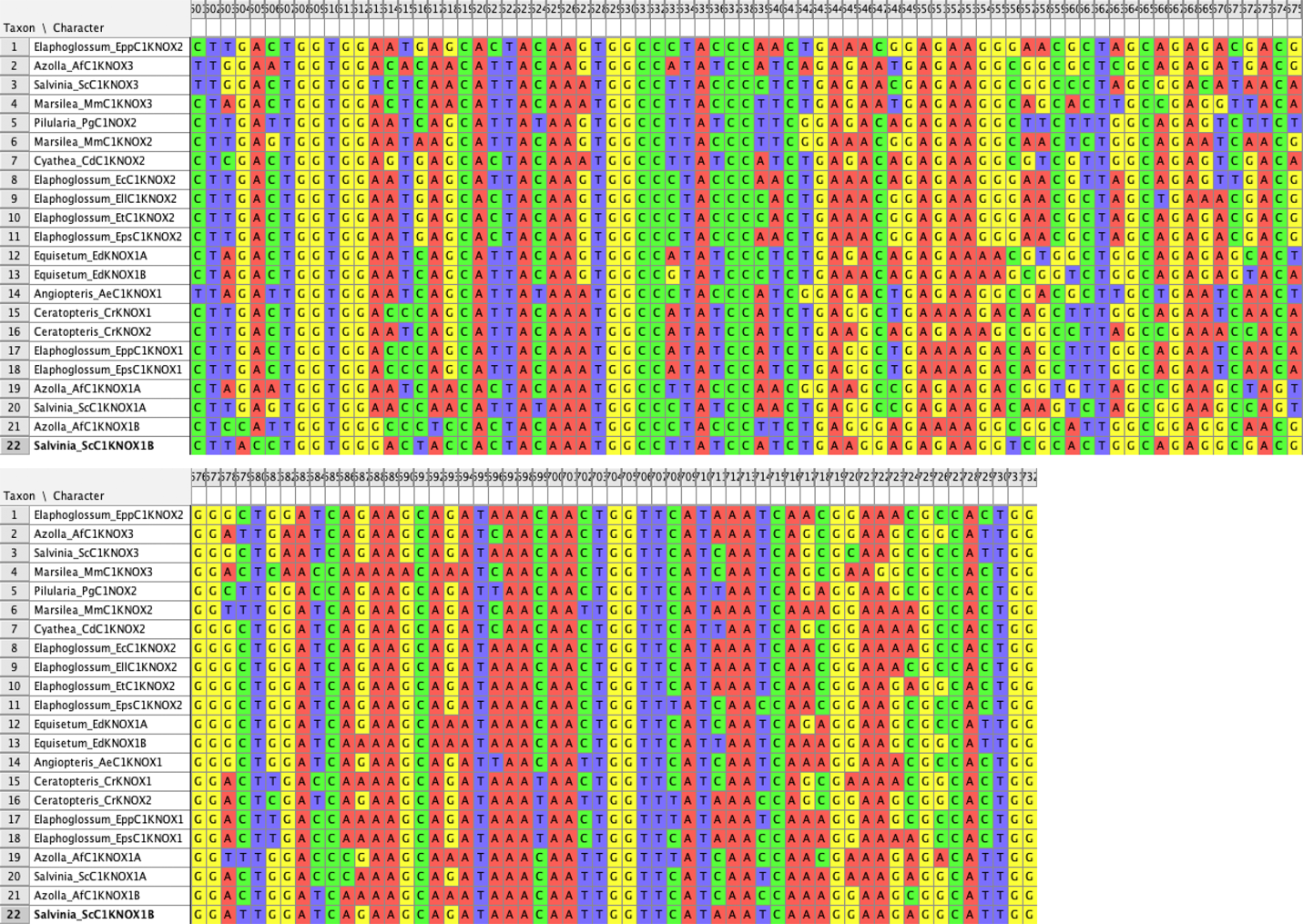


**b.**

**
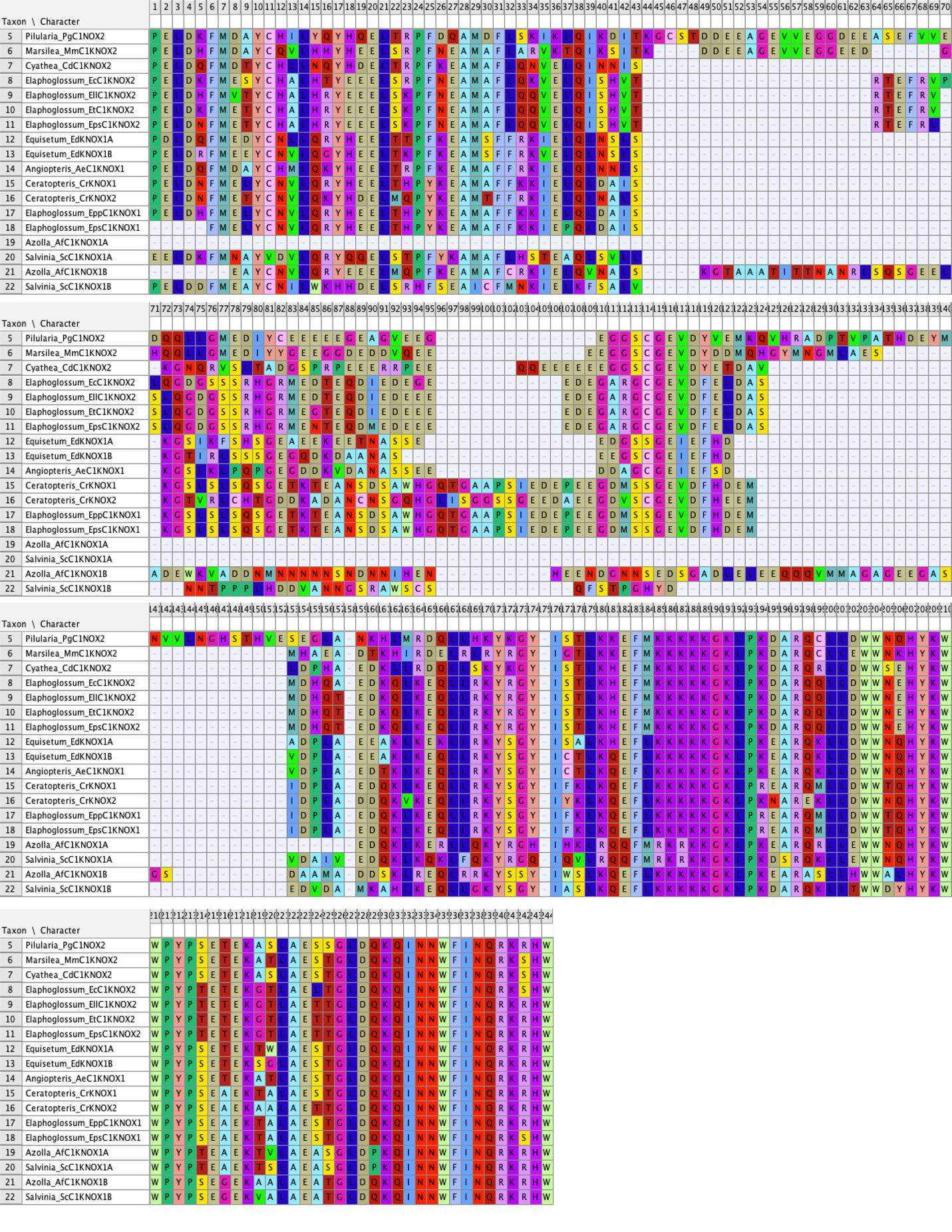
**
