# Supplementary material for: Simple and Divided Leaves in Ferns: Exploring the Genetic Basis for Leaf Morphology Differences in the Genus Elaphoglossum (Dryopteridaceae)
Source: Int J Mol Sci. 2020 Jul 22;21(15):5180. doi: 10.3390/ijms21155180 (PMC7432805; doi:10.3390/ijms21155180)

Vasco and Ambrose—International Journal of Molecular Sciences– Figure S3

**Figure S3.** Additional expression of *Class I KNOX* genes during leaf development in species of the fern genus *Elaphoglossum* with simple and divided leaves. (a,b) *Elaphoglossum peltatum* f. *standleyi* (simple leaves), expression of *EpsC1KNOX1,* longitudinal sections through the SAM and/or leaf primordia. (a) Expression throughout the entire apical dome of the SAM and procambium, including expression in the incipient leaf primordium (Stage 0). (b) Expression throughout the entire young leaf primordium including the leaf apical initial (LAI); expression in the root apical meristem (RAM). (c-g) *Elaphoglossum peltatum* f. *peltatum* (divided leaves). (c-f) Expression patterns of *EppC1KNOX1,* longitudinal sections through the SAM and/or leaf primordia. (c) Expression throughout the entire apical dome of the SAM and procambium, including expression in the incipient leaf primordium (Stage 0). (d) Expression throughout the entire young leaf primordium including the LAI. (e) Expression throughout the entire apical dome of the SAM and procambium; expression in the root apical meristem (RAM). (f) Expression throughout the entire apical dome of the SAM and procambium, including expression in the incipient leaf primordium; expression in the apical region of an older leaf primordium (right). (e) Expression of *EppC2KNOX1* in the leaf primordium, procambium and root primordium. Black Arrowheads, leaf primordia; Brackets, LAI; Stars, SAM; White arrows=root primordium; White arrowheads, procambium; Bars=40 um.


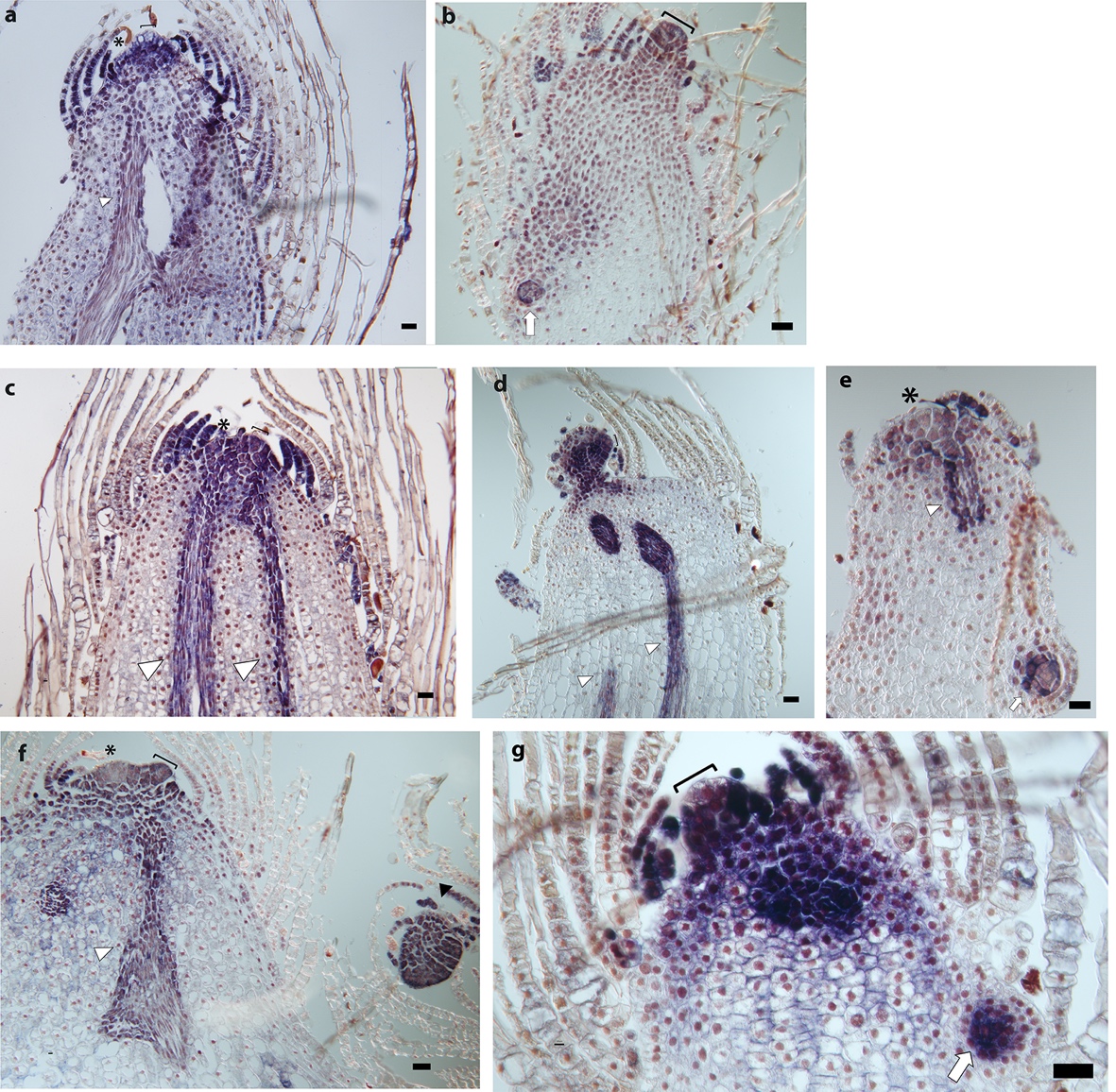

Supplement: Supplementary file 1 [file ijms-21-05180-s001.zip › Vasco&Ambrose_FigureS3_June26.docx]
