# Supplementary material for: Simple and Divided Leaves in Ferns: Exploring the Genetic Basis for Leaf Morphology Differences in the Genus Elaphoglossum (Dryopteridaceae)
Source: Int J Mol Sci. 2020 Jul 22;21(15):5180. doi: 10.3390/ijms21155180 (PMC7432805; doi:10.3390/ijms21155180)
Supplement: Supplementary file 1 [file ijms-21-05180-s001.zip › Vasco&Ambrose_TableS2.docx]

Vasco and Ambrose—International Journal of Molecular Sciences– Table S2

Table S2. Forward and reverse primers designed for in-situ hybridizations.

| **Description** | **Primer F** | **Primer R** | **Probe size bp** |
| --- | --- | --- | --- |
| *Elaphoglossum lloense* Histone H4 | ATGTCAGGCCGGGGTAAGGGAGGC | GGTACTGCCTTGACGCTTGAG | 290 |
| *Elaphoglossum peltatum*  f. *peltatum* Histone H4 | ATGTCTGGCAGAGGTAAGGGAGG | GAGAGTCCGGCCTTGACGCTTCAG | 290 |
| *Elaphoglossum peltatum* f. *standleyi* Histone H4 | TCAGGACGGGGTAAGGGAGGAAG | AAGGGTCCTGCCTTGACGCTTCAG | 290 |
| *Elaphoglossum peltatum*  f. *peltatum* C1Knox Copy 1 | GCAATGTGCTACAGAGGTATCACG | CCTGTTTGAGTTTGAATATGTAGCC | 336 |
| *Elaphoglossum peltatum* f. *standleyi* C1Knox Copy 1 | GCAATGTGTTACAGAGGTATCATG | CCTGTTTGAGTTTGAATATGTAGCC | 336 |
| *Elaphoglossum lloense* C1Knox Copy 2 | GACCTATTGCCATGCTCTTCA | CATGAATTCATGTTTGAGGGTAC | 340 |
| *Elaphoglossum peltatum*  f. *peltatum* C1Knox Copy 2 | GACCTATTGCCATGCTCTTCA | CATGAATTCATGTTTGAGGGTAC | 340 |
| *Elaphoglossum peltatum* f. *standleyi* C1Knox Copy 2 | GACCTATTGCCATGCTCTTCA | CATGAATTCATGTTTGAGGGTAC | 340 |
